# linc00968 inhibits the tumorigenesis and metastasis of lung adenocarcinoma via serving as a ceRNA against miR-9-5p and increasing CPEB3

**DOI:** 10.18632/aging.103833

**Published:** 2020-11-05

**Authors:** Huaping Tang, Xiaolei Han, Yan Feng, Yueqin Hao

**Affiliations:** 1 Department of Pulmonary and Critical Care Medicine, Qingdao Municipal Hospital, Qingdao, Shandong, China; 2 Health Office, Qingdao Municipal Hospital, Qingdao, Shandong, China

**Keywords:** linc00968, miR-9-5p, CPEB3, LUAD

## Abstract

Increasing evidence confirms that long noncoding RNAs (lncRNAs) exert vital functions in multiple biological process among malignant cancers. In the current study, we uncovered that linc00968 was downregulated in lung adenocarcinoma (LUAD). Furthermore, the low level of linc00968 was correlated with worse prognosis in patients with LUAD. Upregulation of linc00968 restrained the growth and metastatic phenotypes of LUAD cell *in vitro* and *in vivo*. Using bioinformation methods and luciferase reporter assay, we identified that linc00968 acted as a competing endogenous RNA (ceRNA) via sponging miR-9-5p to modulate the level of Cytoplasmic Polyadenylation Element Binding Protein 3 (CPEB3) in LUAD. In addition, LUAD cell migration, colony formation and epithelial-mesenchymal transition (EMT) process were suppressed by linc00968 while these aggressive traits were reversed by miR-142-5p or CPEB3 silencing. Altogether, our work disclosed that linc00968 played a critical role in LUAD and linc00968/miR-9-5p/CPEB3 regulatory axis might be a potential treatment target in LUAD.

## INTRODUCTION

Lung cancer is the major cause of cancer-associated death worldwide. Nearly 80% of lung cancer diagnoses are non-small-cell lung cancer (NSCLC) and the major subtypes are lung squamous cell carcinoma (LSCC) and lung adenocarcinoma (LUAD) [1]. Despite the great advances in the diagnostics, targeted drugs and immunotherapy, the overall survival (OS) remains unsatisfactory for patients with NSCLC owing to patients diagnosed with NSCLC had cancer cell diffusion and metastases [2]. A large amount of genetic alterations have been proved to be “drivers” in the progression of NSCLC, including mesenchymal-epidermal transition (EMT)-related genes and mutations in epidermal growth factor receptor (EGFR) [3, 4]. However, the mechanisms behind NSCLC development are not well understood. Thus, elaboration of the mechanisms behind the metastasis of NSCLC is crucial to improvement the survival rates of patients.

Accumulating reports have disclosed that lncRNAs are critical regulators that affect the tumorigenesis and progression of cancers through modulating multiple aspects of cancer cell behavior, such as cell growth, apoptosis, autophagy, and metastasis [5–8]. For instance, lncRNA HUMT hypomethylation facilities the lymphangiogenesis and metastasis of triple-negative breast carcinoma cell via activating FOXK1 transcription [9]. Down-regulation of SBF2-AS1 suppresses the progression of breast carcinoma via sponging miRNA-143 and inhibiting RRS1 [10]. Early report has indicated that linc00968 reduces the migration of breast cancer and tumor angiogenesis by reducing miR-423-5p [11].

Recently, linc00968 identified to be downregulated in breast carcinoma and is also related to poor prognosis in patients [12]. In NSCLC, linc00968 serves as oncogene in lung cancer through activating the Wnt signaling axis [13]. However, after we checked the expression pattern of linc00968 in NSCLC using The Cancer Genome Atlas Program (TCGA), we observed that linc00968 was significantly downregulated in LUAD and LSCC. Meanwhile, in Wang, et al report, the conclusion is contrary to the data from cancer microarray database, Oncomine (https://www.oncomine.org/resource/login.html) [13]. Hence, the expression level of linc00968 and the underlying mechanisms in NSCLC progression needs to be fully illuminated. In competing endogenous RNAs (ceRNAs) network, lncRNAs function as scaffolds with miRNAs, and thus increase the expression of various mRNAs targeted by miRNAs. For instance, lncRNA MALAT1 contributes to sorafenib resistance by targeting miR-140-5p/Aurora-A signaling in hepatocellular carcinoma [14]. LncRNA TUG1 induces the autophagy-associated paclitaxel resistance via sponging miR-29b-3p in ovarian carcinoma [15]. LncRNA-ROR induces EMT and contributes to breast cancer tumorigenesis and metastasis [16]. However, whether linc00968 could regulate the EMT of lung cancer by pairing with miRNAs is still largely unknown.

In the current study, we identified linc00968 was downregulated in LUAD, from published GEO microarray data and our collected LUAD case-cohort study. We further demonstrated that low level of linc00968 was associated with the metastasis and poor survival of patients with LUAD. The loss-and gain function assays revealed that upregulation of linc00968 distinctly restrained the growth and aggressive traits of LUAD cell through sponging miR-9-5p to inhibit the EMT process of LUAD cell.

## RESULTS

### linc00968 predicts the overall survival of patients with LUAD

**Figure 1:**
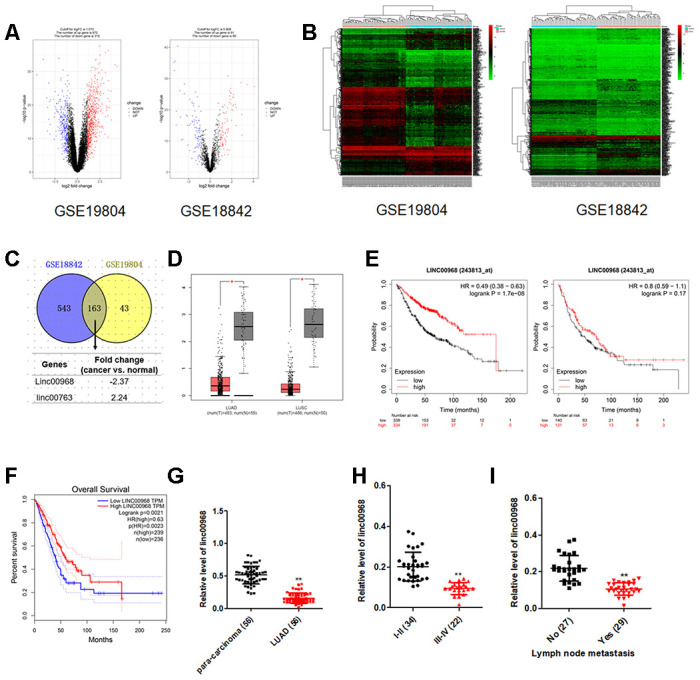
**The expression and clinical significance of linc00968 in NSCLC.** (**A**) Volcano plot showing the differential genes (red dots and blue dots) in the expression microarray. (**B**) Heatmap of GSE19804 and GSE18842. (**C**) Venn diagram showing the common downregulated or upregulated lncRNAs in two GEO dataset. (**D**) linc00968 expression in LUAD and LUSC from TCGA, with red boxplot referring to cancer sample and gray boxplot referring to normal sample. (**E**) Survival analysis of linc00968 in LUAD and LUSC from Kaplan-Meier survival plot. (**F**) Survival analysis of linc00968 in LUAD from TCGA. (**G**) Expression of linc00968 in LUAD tissues and para-carcinoma tissues examined by qRT-PCR assay. ^**^P<0.01 versus the para-carcinoma tissues. (**H**–**I**) The expression levels of linc00968 in LUAD patients with different pathological stages and lymph node metastasis. ^**^P<0.01 versus the I-II or patients with no lymph node metastasis.

Initially, microarray analysis of expression profiles GEO: GSE19804 and GSE18842 displayed that linc00968 was significantly downregulated in NSCLC (Figure 1A–1C). The expression levels of linc00968 in both LUAD and LUSC were confirmed using TCGA dataset (Figure 1D). Using the Kaplan-Meier survival plot (http://kmplot.com/analysis/index.php?p=service&cancer=lung), we revealed that LUAD patients who had high level of linc00968 exhibited better overall survival (OS). However, the dysregulation of linc00968 was irrelevant with the OS of patients with LUSC (Figure 1E). To confirm this observation, the prognostic value of linc00968 in LUAD was further verified using TCGA. As shown in Figure 1F, higher level of linc00968 in patients with LUAD resulted in better survival rate. The levels of linc00968 in LUAD and para-carcinoma tissues from 56 cases of patients with LUAD were detected using qRT-PCR. As shown in Figure 1G–1I, linc00968 was significantly downregulated in LUAD. The dysregulated expression of linc00968 was negatively related to the lymph node metastasis (LNM) and clinical stage. However, linc00968 expression level was irrelevant with age, sex, and tumor size of LUAD (Supplementary Table 1). Taken together, these findings indicate that linc00968 is downregulated in LUAD.

### Effects of linc00968 on lung cancer cell growth, migration and invasion

**Figure 2:**
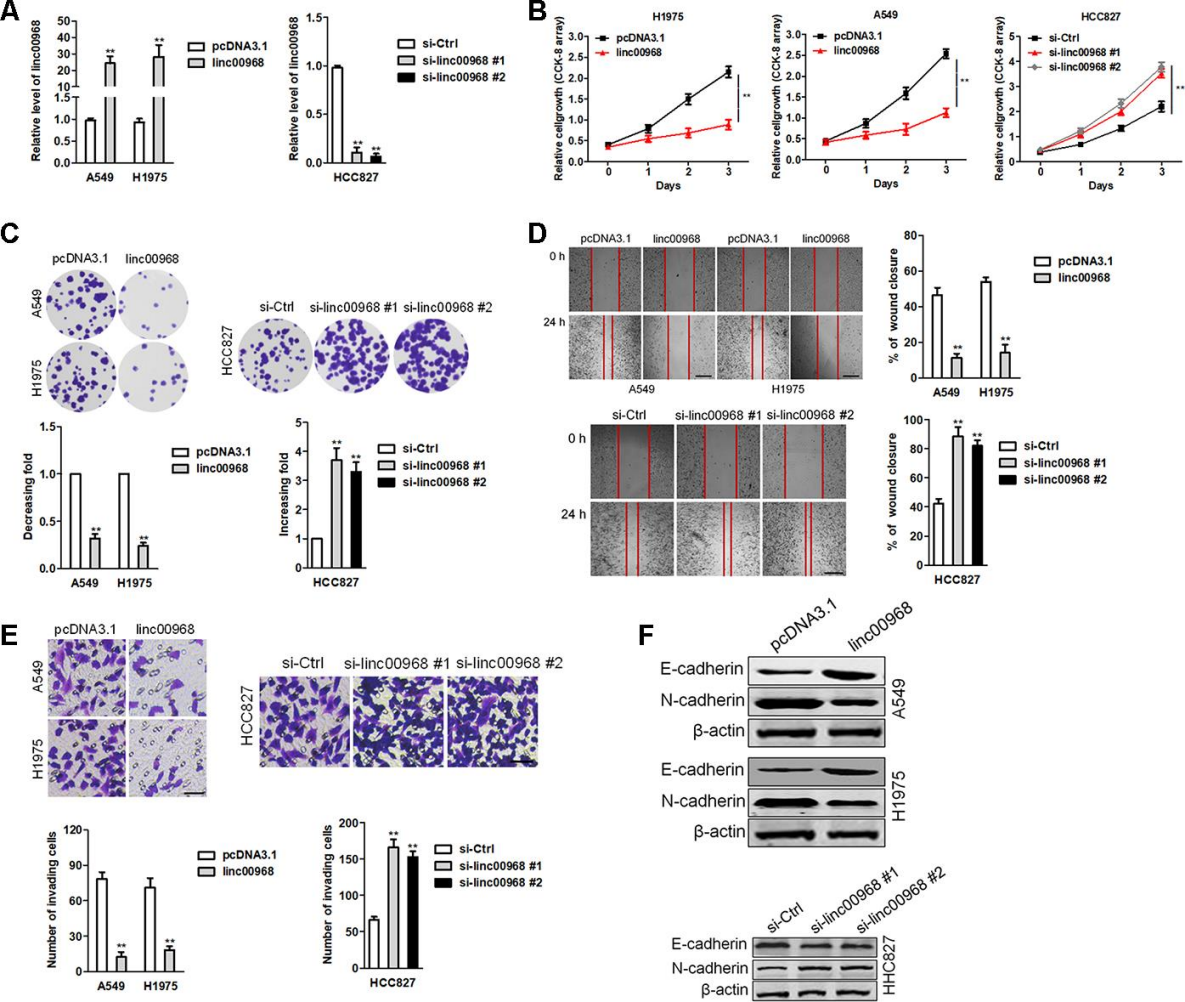
**linc00969 inhibits LUAD cell growth.** (**A**) The levels of linc00968 in linc00968 overexpressing A549 and H1975 cell or linc00968 knockdown in HCC827 were detected with qRT-PCR assay, (**B**) CCK-8 assays showed that overexpression of linc00968 inhibited cell proliferation in A549 and H1975 cell, whereas knockdown of linc00968 increased cell proliferation in HCC827 cell. (**C**) Colony-forming assays showed that linc00968 reduced A549 and H1975 colony formation, whereas knockdown of linc00968 enhanced the colony formation of HCC827 cell. (**D**) Representative images and the percentage of migration in wound healing assay. (**E**) Representative images and the number of invading cells in Transwell invasion assay. (**F**) The expressions of E-cadherin and N-cadherin were detected using western blotting assay. ^**^P<0.01 versus pcDNA3.1 or si-Ctrl.

To elaborate the roles of linc00968 in LUAD, we firstly determined the baseline levels of linc00968 in three LUAD cell lines. H1975 and A549 cell lines were used for overexpression and HCC827 cell line was selected for knockdown of linc00968 (Supplementary Figure 1). The efficiencies of interference and overexpression were certified by qRT-PCR (Figure 2A). Then, we analyzed the function of linc00968 in LUAD cell aggressiveness. The results implied that upregulation of linc00968 repressed the growth of A549 and H1975 cell, whereas linc00968 silencing increased HCC827 cell proliferation as determined by CCK-8 assay (Figure 2B) and colony formation assay (Figure 2C). Similarly, linc00968 impaired the migration abilities of A549 and H1975 cell, whereas silencing of linc00968 reinforced the mobility of HCC827 cell (Figure 2D). Additionally, transfection of linc00968 reduced the invasion abilities of A549 and H1975 cell, whereas si-linc00968 promoted HCC827 cell invasiveness (Figure 2E). Moreover, upregulation of linc00968 raised the expression of E-cadherin and reduced the expression of N-cadherin in A549 and H1975 cell as demonstrated by western blotting. Nevertheless, si-linc00968 transfection caused opposite results in the expressions of EMT-related markers in HCC827 cell (Figure 2F). Altogether, these data suggest that linc00968 regulates the metastatic-related traits of LUAD cell.

### linc00968 serves as a ceRNA and binds to miR-9-5p

**Figure 3:**
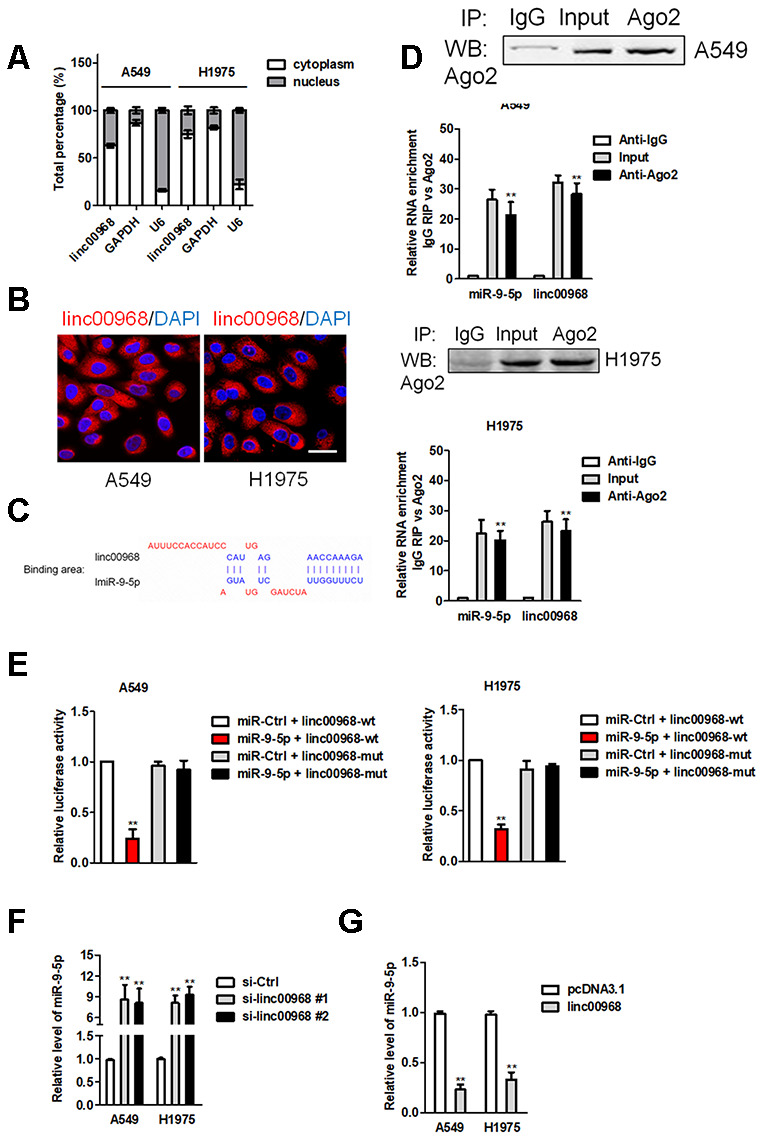
**linc00968 acts as a ceRNA to sponge miR-9-5p.** (**A**) qRT-PCR analysis of linc00968 expression in A549 and H1975 cell. (**B**) RNA-FISH showed the subcellular localization of linc00968 in A549 and H1975 cell. linc00968 was stained with Cy3 (green) and nuclei were stained with DAPI (blue). (**C**) Prediction of miR-9-5p binding sites in the linc00968. (**D**) Ago2-RIP was performed in A549 and H1975 cell. (**E**) Luciferase activity in A549 and H1975 cell cotransfected with wt/mut linc00968 plasmid, and miR-9-5p mimic or miR-Ctrl. (**F**) Relative levels of miR-9-5p in A549 and H1975 cell transfected with si-linc00968 or scrambled control. (**G**) Relative levels of miR-9-5p in A549 and H1975 cell transfected with pcDNA3.1 or linc00968. ^**^P<0.01 versus pcDNA3.1 or si-Ctrl.

Then, we measured the localization of linc00968 in LUAD cell and observed that linc00968 was mainly localized in the cytoplasm of A549 and H1975 cell (Figure 3A). Similar results were obtained in FISH assay (Figure 3B). Using the human lncRNA targets prediction tool LncBase Predicted v.2, we predicted linc00968 could be targeted by several miRNAs. Among them, miR-9-5p (Figure 3C) has been proved to be involved in regulating the progression of caners [17–19]. Next, we determined the correlation between linc00968 and miR-9-5p using RNA RIP assay. As shown in Figure 3D, the Ago2 protein was distinctly immunoprecipitated from A549 or H1975 cell extracts, and both miR-9-5p and linc00968 were remarkedly enriched in anti-Ago2 group when compared with the anti-IgG group. Luciferase reporter gene assay suggested that transfected of miR-9-5p decreased the luciferase activity of linc00968-wt but had no suppressive impact on linc00968-mut (Figure 3E). Moreover, linc00968 silencing raised the levels of miR-9-5p in A549 and H1975 cell (Figure 3F). Meanwhile, upregulation of linc00968 markedly degraded the expression of miR-9-5p (Figure 3G). These findings indicate that linc00968 acts as a ceRNA and binds to miR-9-5p.

### miR-9-5p exerts an oncogenic role in LUAD

**Figure 4:**
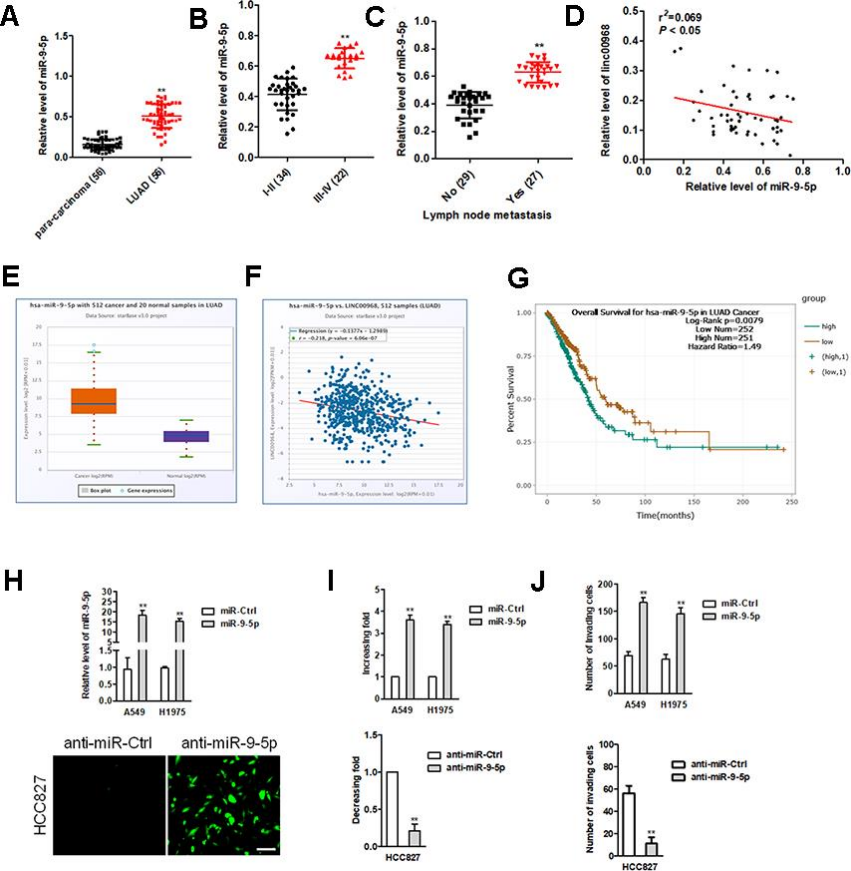
**miR-9-5p is highly expressed in LUAD tissues and exerts oncogenic roles in LUAD cell.** (**A**) Relative expression levels of miR-9-5p in LUAD tissues were measured by qRT-PCR. (**B**, **C**) The expression levels of miR-9-5p in LUAD patients with different pathological stages and lymph node metastasis. ^**^P<0.01 versus the I-II or patients with no lymph node metastasis. (**D**) Correlation analysis of linc00968 and miR-9-5p in 56 cases of LUAD tissues. (**E**) miR-9-5p expression in LUAD from TCGA. (**F**) Correlation analysis of linc00968 and miR-9-5p in TCGA. (**G**) Survival analysis of miR-9-5p in LUAD from TCGA. (**H**) miR-9-5p mimics was transfected A549 and H1975 cell. anti-miR-9-5p was transfected into HCC827 cell. The transfection efficiency was detected using qRT-PCR and immunofluorescence. (**I**, **J**) The colony formation and invasion abilities of LUAD cell transfected with miR-9-5p mimics or anti-miR-9-5p were measured by colony formation or Transwell invasion assay, respectively. ^**^P<0.01 versus miR-Ctrl.

Early reports have revealed that miR-9-5p is an oncomiR during cancer tumorigenesis [20]. Consistently, miR-9-5p was also overexpressed in LUAD tissues compared to in para-carcinoma tissues (Figure 4A). The higher expression level of miR-9-5p was positively associated with the metastasis and advance stage, while miR-19-5p expression level was irrelevant with age, sex, and tumor size in patients with LUAD (Figure 4B, 4C). Importantly, an inversely relationship between miR-9-5p and linc00968 in LUAD tissues was observed (Figure 4D). In TCGA, miR-9-5p was significantly upregulated in LUAD and negatively associated with linc00968 expression (Figure 4E, 4F). Using the TCGA dataset, we also revealed that patients who had high miR-9-5p expression exhibited poor OS compared with patients had low miR-9-5p expression (Figure 4G). A549 and H1975 cell was transfected with miR-9-5p, while HCC827 cell was transfected with FAM labeled miR-9-5p inhibitor (anti-miR-9-5p) (Figure 4H). Functionally, colony formation and invasive capacities were heightened after A549 and H1975 cell were treated with miR-9-5p mimics (Figure 4I, 4J). In contrast, opposite trends in HCC827 were obtained when cell was treated with anti-miR-9-5p (Figure 4I, 4J).

### linc00968 raises the expression of CPEB3, an endogenous target of miR-9-5p

**Figure 5:**
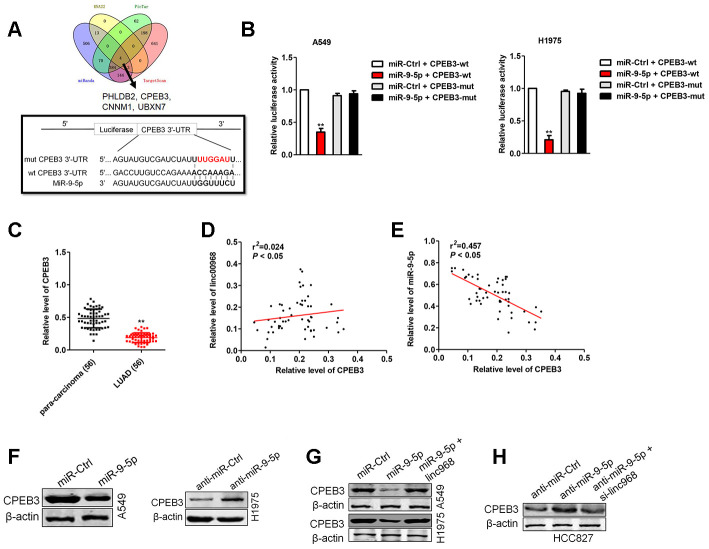
**CPEB3 is a potential target of miR-9-5p.** (**A**). Four bioinformatics methods (RNA22, Targetscan, PicTar and miRanda) were used predicted the targets of miR-9-5p. Prediction of miR-9-5p binding sites in the CPEB3. (**B**) Luciferase activity in A549 and H1975 cell cotransfected with wt/mut CPEB3 plasmid, and miR-9-5p mimic or miR-Ctrl. (**C**) Expression of CPEB3 in LUAD tissues and para-carcinoma tissues examined by qRT-PCR assay. ^**^P<0.01 versus the para-carcinoma tissues. (**D**) Correlation analysis of linc00968 and CPEB3 in 56 cases of LUAD tissues. (**E**) Correlation analysis of CPEB3 and miR-9-5p in 56 cases of LUAD tissues. (**F**) The expression of CPEB3 was detected using western blot in miR-9-5p transfected A549 and anti-miR-9-5p transfected H1975 cell. (**G**) A549 and H1975 cell was transfected miR-9-5p or cotransfected with miR-9-5p and linc00968. (**H**) HCC827 was transfected anti-miR-9-5p or cotransfected with anti-miR-9-5p and si-linc00968. The expression of CPEB3 was assessed by western blot.

To further clarify the molecular mechanisms of linc00968/miR-9-5p in LUAD, we found that PHLDB2, CPEB3, CNNM1 and UBXN7 were the potential downstream genes of miR-9-5p using four bioinformatics methods (RNA22, Targetscan, PicTar and miRanda) (Figure 5A). Among the targets of miR-9-5p, we focused on CPEB3, whose mRNA level was significantly decreased by miR-9-5p in LUAD cell (data not shown). The binding sites between miR-9-5p and CPEB3 3’-UTR were illustrated in Figure 5A. To verify the directly binding between CPEB3 and miR-9-5p, luciferase gene reporter assay was performed. The luciferase activity in CPEB3-wt + miR-9-5p group was strikingly lessened compared with miR-Ctrl group, whereas the luciferase activity in the CPEB3-mut group was not suppressed by miR-9-5p (Figure 5B). We also measured the levels of CPEB3 in BEAS-2B cell and three LUAD cell lines. The mRNA levels of CPEB3 were lower in LUAD cell versus that in BEAS-2B cell (Supplementary Figure 2). Similarly, the levels of CPEB3 were downregulated in LUAD tissues than in para-carcinoma tissues (Figure 5C), resulting in a positive correlation between linc00968 and CPEB3, and a negative relationship between miR-9-5p and CPEB3 (Figure 5D, 5E). Subsequently, we assessed the effects of miR-9-5p on the protein expressions of CPEB3 by western blot, and we observed that the expression of CPEB3 was reduced in miR-9-5p group than that in miR-Ctrl group, whereas CPEB3 expression in anti-miR-9-5p showed the contrary result (Figure 5F). Finally, miR-9-5p reduced the expressions of CPEB3, which was reversed by transfection with linc00968 overexpression plasmid (Figure 5G). On the contrary, anti-miR-9-5p elevated the level of CPEB3, which was reversed after si-linc00968 transfection (Figure 5H). Together, these observations imply that linc00968 regulates the expression of CPEB3 via sponging miR-9-5p.

### linc00968 inhibits EMT process in LUAD cell via sponging miR-9-5p

**Figure 6:**
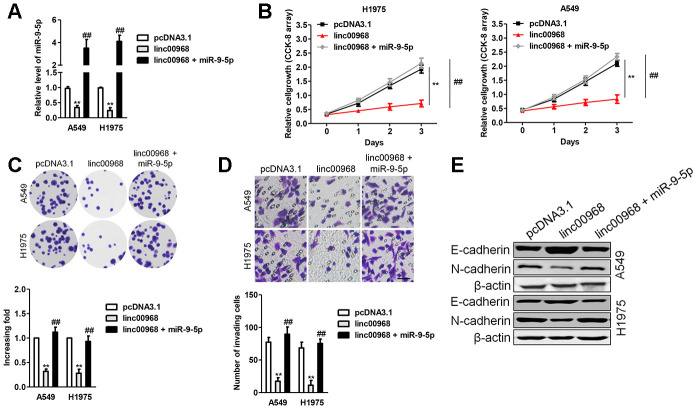
**Overexpression of miR-9-5p reverses the inhibitory roles of linc00968 in LUAD cell.** (**A**) A549 or H1975 was transfected with linc00968 or cotransfected with linc00968 and miR-9-5p. The level of miR-9-5p was detected with qRT-PCR assay. (**B**–**D**) The proliferation, colony formation and invasion abilities were rescued by miR-9-5p. Scale bar, 200 μm. (**E**) The expressions of E-cadherin and N-cadherin were detected by western blot. ^**^P<0.01 versus pcDNA3.1 or linc00968.

To affirm whether linc00968 exerts its impacts on LUAD cell by sponging miR-9-5p, “rescue” experiments were carried out to check the functional interactions of linc00968 and miR-9-5p. We found that after transfected miR-9-5p mimics into linc00968 overexpressing cell, the proliferation, colony formation and invasive capacities were obviously rescued compared with those of LUAD cell transfected with linc00968 alone (Figure 6A–6D). Next, the impacts of linc00968 on the EMT process of A549 and H1975 cell were analyzed. Immunoblotting analysis showed that the level of E-cadherin was upregulated after linc00968 was overexpressed, whereas N-cadherin was reduced after overexpressing linc00968, but this trend was partially abolished by reintroduction of miR-9-5p (Figure 6E).

### Silencing of CPEB3 reverses the effect of linc00968

**Figure 7:**
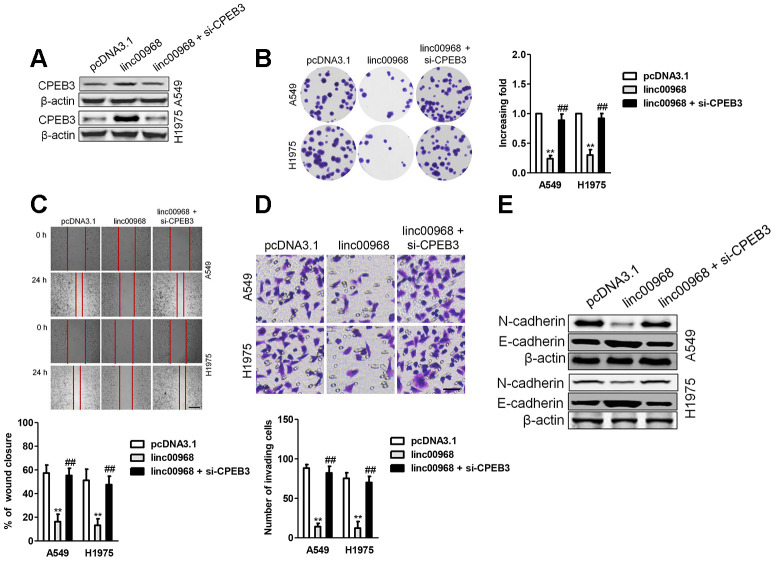
**Downregulation of CPEB3 reverses the inhibitory roles of linc00968 in LUAD cell.** (**A**) A549 or H1975 was transfected with linc00968 or cotransfected with linc00968 and si-CPEB3. The level of CPEB3 was detected with western blot assay. (**B**–**D**) The colony formation, migration and invasion abilities were rescued by si-CPEB3. Scale bar, 200 μm. (**E**) The expressions of E-cadherin and N-cadherin were detected by western blot. ^**^P<0.01 versus pcDNA3.1 or linc00968.

To demonstrate that linc00968 inhibits LUAD cell metastatic-phenotypes in a CPEB3-dependent manner, linc00968 overexpressing A549 and H1975 cell was transfected with siRNA CPEB3 plasmid (si-CPEB3). In A549 and H1975 cell, functional assays *in vitro* indicated that downregulation of CPEB3 rescued the viability and mobility of LUAD cell impaired by linc00968 (Figure 7A–7D). Besides, downregulation of CPEB3 increased the level of N-cadherin that was inhibited by linc00968. On the contrary, knocked-down of CPEB3 abolished the enhancive effect of linc00968 on the expression level of E-cadherin (Figure 7E). Taken together, CPEB3 silencing rescues LUAD cell proliferation, migration, and invasion that were inhibited by linc00968.

### linc00968 suppresses the tumor growth of LUAD cell *in vivo*

**Figure 8:**
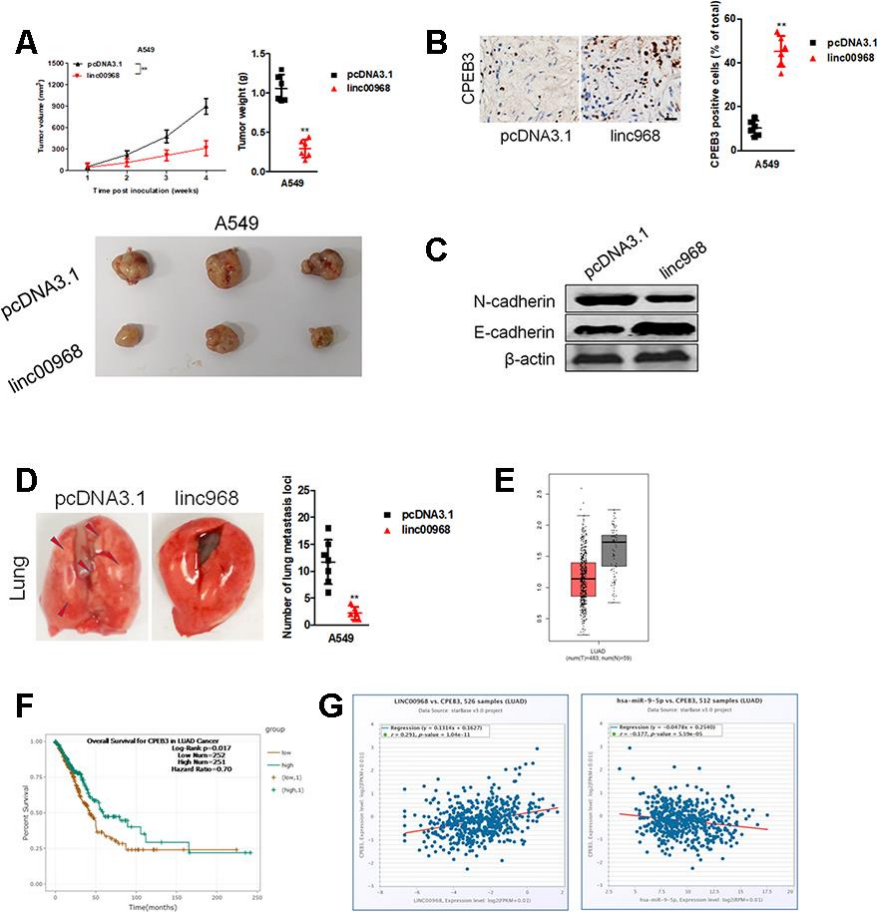
**Overexpression of linc00968 suppresses the growth of A549 cell *in vivo*.** (**A**) The respective xenograft tumors from the nude mice were exhibited. Tumor weights and tumor growth curves were analyzed. (**B**) IHC of CPEB3 in the subcutaneous tumors. Scale bar, 200 μm. (**C**) The expressions of E-cadherin and N-cadherin were detected using western blotting assay. (**D**) Representative photos for lung metastasis nodules. The number of nodules lung metastatic foci as calculated. (**E**) CPEB3 expression in TCGA, with red boxplot referring to LUAD sample and gray boxplot referring to normal sample. (**F**) Survival analysis of CPEB3 in LUAD from TCGA. (**G**) The correlations between CPEB3 and miR-9-5p or linc00968 were analyzed in TCGA. ^**^P<0.01 versus pcDNA3.1.

Finally, pcDNA3.1 or pcDNA3.1 expressing linc00968 stable transfected A549 cells were inoculated into nude mice. As shown in Figure 8A, the tumor volumes and weights in mice injected with linc00968 overexpressing A549 cells were markedly restrained compared with the pcDNA3.1 control group. Moreover, IHC staining displayed that CPEB3 level was raised in the linc00968-overexpressing xenograft tumor tissues (Figure 8B). Furthermore, western blotting analysis of tumor tissues suggested that linc00968 inhibited the EMT-markers *in vivo* (Figure 8C). Next, we detected the influence of linc00968 in the metastasis of A549 cell in experimental metastasis model. Our results revealed that the number of lung metastatic foci was reduced in mice injected with linc00968 overexpressed A549 cell compared with the vector group (Figure 8D). In TCGA, CPEB3 is significantly downregulated in LUAD and patients who had high CPEB3 expression exhibited better OS compared with patients with low CPEB3 expression (Figure 8E, 8F). Finally, an inversely relationship between miR-9-5p and CPEB3 whereas a positively relationship between CPEB3 and linc00968 in LUAD tissues were found (Figure 8G). These findings further verify the functions of linc00968 in LUAD cell proliferation and metastasis *in vivo*.

## DISCUSSION

LUAD is a malignant disease with unsatisfactory curative effects in the clinical practice owing to a high prevalence and poor prognosis. Dysregulated cancer-associated genes play critical actions in the development of LUAD [21–24]. Recent evidences have highlighted the functions of lncRNAs in the tumorigenesis and development of cancers. Numerous lncRNAs have been proved to be dysregulated and regulate the expressions of target genes through multiple mechanisms in LUAD [25, 26]. For instance, lncRNA SNHG7 is downregulated in LUAD tissues and altered SNHG7 expression induces the changes in LUAD cell proliferation and migration [27]. LncRNA HMMR-AS1 promotes the proliferation and metastasis of LUAD cells by regulating miR-138/sirt6 axis [28]. Recently, increasing studies elucidate that aberrantly expressed linc00968 play vital roles in the tumorigenesis of cancers through involving to diverse biological behaviors [11]. However, the function and mechanisms of linc00968 in LUAD need to be more extensively illuminated.

To disclose the functions of linc00968 in human LUAD, we analyzed the level of linc00968 in LUAD tissue using GEO dataset and TCGA database. The results showed that linc00968 was downregulated in LUAD compared with in matched normal tissue. Additional, low expression level of linc00968 in was significantly correlated with the poor clinical outcome of patients with LUAD, which indicated that linc00968 might be a potential predictor for poor OS of LUAD. To analyze the function of linc00968 in LUAD cell, we constructed linc00969 knockdown and overexpression cell line. Cancer cell metastasis is the major cause for the mortality in patients with LUAD, which urges us to investigate the molecular mechanisms underlying cancer cell metastasis. As a result, upregulation of linc00968 distinctly repressed the growth, migration ability and invasiveness of A549 and H1975 cell whereas downregulation of linc00968 caused opposite trends in HCC827 cell.

EMT is a complicated process in which epithelial cancer cell lose cell-cell adhesion and obtain aggressive properties to become mesenchymal cell [29]. Then, we measured the expressions of EMT-related markers (N-cadherin and E-cadherin) in LUAD cell and found that linc00968 silencing decreased the expression of E-cadherin and raised the expression of N-cadherin. Next, bioinformatics algorithms were selected to find the potential binding miRNAs of linc00968 in LUAD. Then, we chose miR-9-5p in our further study. MiR-9-5p is identified to be overexpressed in multiple cancers, including ovarian cancer, gastric carcinoma, osteosarcoma and NSCLC. In our study, we observed that linc00968 shared complementary binding sites with miR-9-5p and upregulation of decreased miR-9-5p level. Furthermore, linc00968 and miR-9-5p could be pulled down by anti-Ago2. Altogether, these results implied that linc00968 served as a ceRNA to bind with miR-9-5p.

MiRNAs bind to the 3’-UTR of their target mRNAs and cause the repression of translation or the degradation of genes. To explore the precise functions of linc00968/miR-9-5p axis, we sought the downstream targets of miR-9-5p using bioinformatics methods. We observed that miR-9-5p might target CPEB3 via binding to its 3′-UTR. Moreover, the level miR-9-5p was negatively associated with CPEB3 level in LUAD samples, and we further demonstrated that miR-9-5p transfection decreased the expression of CPEB3 in LUAD cells *in vitro*. Furthermore, we revealed that linc00968 could reverse the impact of miR-9-5p on the expression of CPEB3 in LUAD cell. Meanwhile, addition of miR-9-5p reversed the suppressive effects of linc00968 on the growth and invasion of LUAD cell. However, CPEB3 knockdown reversed the suppressive influences of linc00968 on the growth and invasiveness of LUAD cell, which certificated the existence of linc00968/miR-9-5p/CPEB3 axis in LUAD.

This study has several potential limitations. One limitation is that we only analyzed the expression level of linc00968 and its functions in LUAD tissues. The precise role of linc00968 in squamous cell carcinoma needs to be explored in future. Our experimental approach was designed to prove that linc00968 is a sponge for miR-9-5p *in vitro*. However, another xenograft tumor model needs to be constructed using linc00968 and miR-9-5p cotransfected LUAD cells. Another potential limitation is that we have not attempted to detect the colocalization of linc00968 and miR-9-5p by RNA-FISH in LUAD cells. In summary, we identified that linc00968 was downregulated in LUAD and decreased linc00968 expression was associated with the worse outcomes in patients with LUAD. Moreover, we testified that linc00968 inhibited the growth and invasion of LUAD cells *in vitro* and suppressed tumor growth and metastasis *in vivo* via intervening the miR-9-5p/CPEB3 axis.

## MATERIALS AND METHODS

### Tissue collection and cell culture

56 cases of LUAD tissues and adjacent noncancerous specimens were collected from patients with LUAD from Qingdao Municipal Hospital between 2010 and 2016. Tissues were frozen in liquid nitrogen and stored at -80°C. The experiments were approved by the Ethics Committee of Qingdao Municipal Hospital. Informed consent was obtained from patients before this study. The LUAD cell lines A549, H1975 and HCC827, and the immortalized bronchial epithelial cell line, BEAS-2B were bought from Jiangsu Keygenbio Biotechnology Co., Ltd (Naming, Jiangsu, China). Cells were maintained in RPMI-1640 medium (Thermo Fisher Scientific, Waltham, MA, USA) supplemented with 10% FBS and 1% penicillin/streptomycin (Thermo Fisher Scientific) in a humidified atmosphere of 5% CO_2_ at 37 °C.

### Fluorescence *in situ* hybridization (FISH)

A549 or H1975 cells were seeded in confocal dishes for 24 hours and were fixed, prehybridized, and hybridized in hybridization buffer withlinc00968 oligodeoxynucleotide probe (GenePharma, Shanghai, China) for overnight. The signal of the probe was measured using a Fluorescent ISH kit (GenePharma, China). Nuclei in cells were stained by DAPI. The images were detected under confocal microscope.

### Plasmid construction and transfection

Effective siRNA oligonucleotides that targeted linc00968 or CPEB3 as well as negative control siRNAs (si-Ctrl), miR-9-5p mimic and negative control (miR-Ctrl mimic), FAM labeled miR-9-5p inhibitor (anti-miR-9-5p) and negative control (anti-miR-Ctrl) were purchased from RiboBio Co., Ltd (Ribobio, Guangzhou, China). All the oligonucleotides and plasmids were transfected into A549, H1975 or HCC827 cell using Lipofectamine 3000 (Invitrogen, Carlsbad, CA, USA) according to the manufacturer’s instructions. The full-length of linc00968 was synthesized by RiboBio (Guangzhou, China) and subcloned into the pcDNA3.1 (+) vector (Thermo Fisher Scientific, Waltham, MA, USA), according to the manufacturer's instructions.

### qRT-PCR assay

Total RNAs were extracted using RNAiso Plus (TaKaRa, Japan). Reverse transcription was conducted using a Prime Script RT Master Mix kit (TaKaRa) for linc00968. For miR-9-5p, cDNA was synthesized using a miRNA First Strand cDNA Synthesis Kit (Sangon Biotech, Shanghai, China). Then, the cDNA was subjected to real-time PCR on a Quantstudio™ DX system (Applied Biosystems). The level of miR-9-5p was normalized to U6. GAPDH was used as the endogenous control. The relative expressions of genes were calculated using the 2^-ΔΔCT^ method. The primers were listed in Supplementary Table 2.

### Cell proliferation assay

Cells (1 × 10^3^) were cultured in 96-well plates, and cell viability was assessed using a CCK-8 kit (Bioworld, Nanjing, China). The OD value was detected at 490 nm under spectrophotometer at 0 h, 24 h, 48 h, 96 h, respectively. For colony formation assay, cells (500 cells per well) were plated into 6-well plates. Cells were maintained for two weeks, cell colonies were fixed by 4% paraformaldehyde and stained with 1% crystal violet (Sigma, Shanghai, China).

### Cell migration and invasion

Cells were cultured into six well plates, and an artificial wound was made using a 100 μl pipette tip after 24 hours. The images of wounds were captured at 0 hour or 24 hours under an inverted microscope. In invasion assay, a 24-well Transwell chamber (Costar, USA) precoated with Matrigel (BD) was used to measure cell invasion. 0.1 ml cells suspended (3×10^4^) were seeded into the upper chambers and maintained with serum-free medium. Lower chambers were filled with medium containing with 20% FBS. After 24 hours, the invading cells were fixed with 4% paraformaldehyde and stained by 1% crystal violet. The invading cells were counted in five randomly selected fields.

### Luciferase reporter assay

Reporter plasmid with the wild type or mutant type miR-9-5p seed sequences for linc00968 (wt-linc00968 and mut-linc00968) or CPEB3 (wt-CPEB3 and mut-CPEB3) were constructed by GenePharma Co., Ltd (GenePharma, Shanghai, China). When A549 or H1975 cell grew to 80% confluence, the reporter plasmids were cotransfected with miR-9-5p mimics or miR-Ctrl into cells using the Lipofectamine 3000 (Thermo Fisher Scientific), respectively. After 48 hours, the luciferase activity in each group was assessed using Luciferase Reporter Assay System (Promega, Madison, WI, USA).

### Immunoblotting

Proteins were lysed from tumor tissues or LUAD cells using RIPA buffer (CWBIO, Beijing, China). After separated by 10% SDS-PAGE, proteins were transferred onto PVDF membranes and incubated with primary CPEB3 (1:1000, Abcam, Cambridge, UK), N-cadherin (1:1000, Abcam, Cambridge, UK), E-cadherin (1:1000, Abcam, Cambridge, UK), or β-actin (1:1000, Bioworld, China) at 4°C overnight. After that, PVDF membranes were incubated with HRP-conjugated secondary antibody for 2 h. Bands were measured using an ECL kit (Millipore, Braunschweig, Germany).

### RNA immunoprecipitation (RIP)

A magna RIP kit (Millipore, Braunschweig, Germany) was used for RIP assay according to the manufacturer’s protocol. Cell lysate were incubated with RIP immunoprecipitation buffer containing magnetic beads conjugated with Ago2 antibody (Abcam, Cambridge, MA, USA) and anti-IgG (Abcam, Cambridge, MA, USA). Then, Co-precipitated RNAs were collected and detected using qRT-PCR.

### Animal experiments

Animal experiments were approved by the Animal Ethics Committee of Qingdao Municipal Hospital. linc00968-overexpressing or pcDNA3.1 (+) vector stable expressed A549 cells (5 × 10^6^) were subcutaneously injected into BALB/c nude mice. The length and width of tumors were measured using a caliper per week. The tumor volume = 0.5×(length×width^2^). Four weeks later, the mice were sacrificed, and the excised tumor tissues were further used for western blotting and immunohistochemical (IHC) staining for CPEB3. In lung metastasis experiment, 1×10^6^ linc00968-overexpressing or pcDNA3.1 (+) vector stable expressed A549 cells were injected into nude mice via lateral tail veins. Five weeks after injection, the animals were sacrificed, and the number of metastatic foci on the surface of lung was counted.

### Statistical analysis

The Data are presented as the Mean ± SD. Statistical analyses were carried out using GraphPad Prism 7.0. The overall survival rates of patients with LUAD were calculated using Kaplan-Meier method and log-rank test. Two-tailed Student’s t-test or Mann-Whitney U-test were used to determine statistically significant differences between two groups, as appropriate. *P*<0.05 was considered statistically significant.

## Supplementary Material

Supplementary Figures

Supplementary Tables
