# Supplementary material for: linc00968 inhibits the tumorigenesis and metastasis of lung adenocarcinoma via serving as a ceRNA against miR-9-5p and increasing CPEB3
Source: Aging (Albany NY). 2020 Nov 5;12(22):22582–98. doi: 10.18632/aging.103833 (PMC7746359; doi:10.18632/aging.103833)
Supplement: Supplementary Figures [file aging-12-103833-s001..pdf]

SUPPLEMENTARY FIGURES

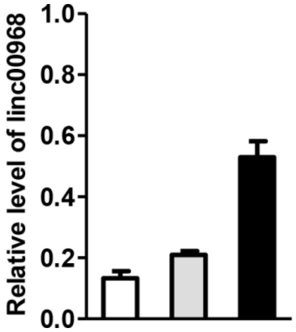

Supplementary Figure 1. qRT-PCR result shown the baseline levels of linc00968 in LUAD cell (A549, H1975 and HCC827).

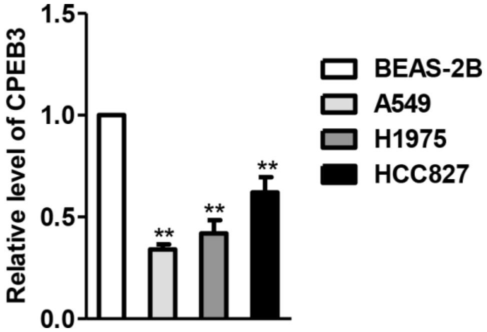

Supplementary Figure 2. qRT-PCR result shown the baseline levels of miR-9-5p in LUAD cell (A549, H1975 and HCC827) and immortalized bronchial epithelial cell line, BEAS-2B.
