# Supplementary material for: linc00968 inhibits the tumorigenesis and metastasis of lung adenocarcinoma via serving as a ceRNA against miR-9-5p and increasing CPEB3
Source: Aging (Albany NY). 2020 Nov 5;12(22):22582–98. doi: 10.18632/aging.103833 (PMC7746359; doi:10.18632/aging.103833)
Supplement: Supplementary Tables [file aging-12-103833-s002..pdf]

SUPPLEMENTARY TABLES

Supplementary Table 1. The association between linc00968, miR-9-5p or CPEB3 expression and clinic-pathological factors in patients with LUAD.

Clinical parameter	linc00968		*P*-value	miR-9-5p		*P-value*	CPEB3		*P*-value
	High	Low		High	Low		High	Low	
**Age (years)**			0.114			0.083			0.042
≤65	12	8		6	14		9	11	
>65	19	17		22	14		20	16	
**Sex**			0.318			0.225			0.104
Male	22	18		22	18		23	17	
Female	9	7		6	10		6	10	
**Size (cm)**			0.31			0.451			0.331
≥3	17	15		15	17		16	16	
<3	14	10		13	11		13	11	
**Differentiation**			0.094			0.619			0.407
Well, Moderate	18	13		15	16		16	15	
Poor	13	12		13	12		13	12	
**Metastasis**			<0.01			<0.01			<0.01
Yes	10	19		21	8		6	23	
No	21	6		7	20		23	4	
**TNM stage**			<0.01			<0.01			<0.01
I-II	27	7		8	26		22	12	
III-IV	4	18		20	2		7	15	

Supplementary Table 2. List of primer sequences were used in the qRT-PCR assay.

Name	Primer	Sequence (5′–3′)
U6	Forward	5′-CTCGCTTCGGCAGCACA-3′
	Reverse	5′-AACGCTTCACGAATTTGCGT-3′
GAPDH	Forward	5′-GGAGCGAGATCCCTCCAAAAT-3′
	Reverse	5′-GGCTGTTGTCATACTTCTCATGG-3′
CPEB3	Forward	5′-CTGCAGCATGGAGAGATAG-3′
	Reverse	5′-GGGTCAGAATGAACAGAAGAG-3′
miR-9-5p	Forward	5′-GTGCAGGGTCCGAGGT-3′
	Reverse	5′-GCGCTCTTTGGTTATCTAGC-3′
